# Reintervention using cholangioscopy for metallic stent obstruction following endoscopic ultrasound-guided hepaticogastrostomy

**DOI:** 10.1055/a-2686-3338

**Published:** 2025-09-11

**Authors:** Yuta Sumida, Seiji Fujigaki, Hiroki Uekado, Hideto Sugao, Misaki Yokoi, Katsuhide Tanaka, Tsuyoshi Sanuki

**Affiliations:** 1 610622 Department of Gastroenterology, Hyogo Prefectural Harima-Himeji General Medical Center, Hyogo, Japan

Endoscopic ultrasound-guided hepaticogastrostomy (EUS-HGS) is a valuable alternative for patients for whom endoscopic retrograde cholangiopancreatography (ERCP) is not feasible. However, recurrent biliary obstruction occurs in approximately 19.1–33% of cases, often necessitating reintervention 1 2 . Herein, we report a case of successful reintervention using peroral cholangioscopy to treat biliary obstruction caused by tissue hyperplasia at the uncovered portion of a self-expandable metallic stent (SEMS) placed during EUS-HGS ( Video 1 ).

Reintervention using cholangioscopy for metallic stent obstruction following endoscopic ultrasound-guided hepaticogastrostomy.Video 1

A 75-year-old man who underwent EUS-HGS for duodenal papillary carcinoma ( Fig. 1 ) was diagnosed with acute cholangitis due to stent dysfunction. Reintervention was attempted using the previously created endosonographic route.

**Fig. 1:**
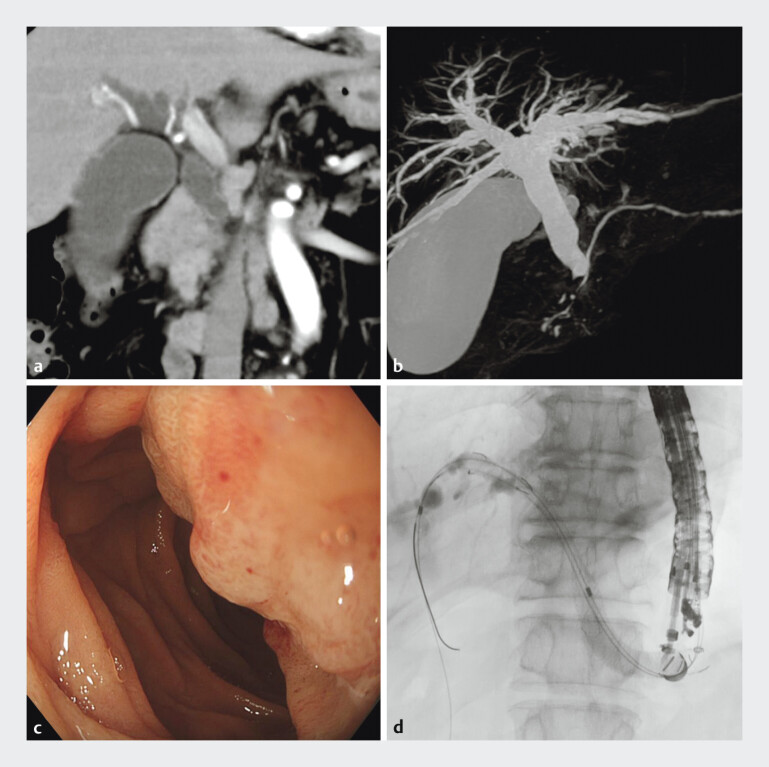
**a** Computed tomography (CT) image showing duodenal papillary carcinoma. **b** Magnetic resonance cholangiopancreatography (MRCP) showing a distal bile duct stricture. **c** Gastroscopy showing duodenal stenosis due to duodenal papillary cancer. **d** A partially covered self-expandable metallic stent was placed on the B3 branch.

A dual-channel endoscope (GIF-2TQ260M, Olympus, Japan) was used to trim the SEMS. The SEMS was grasped with forceps and trimmed via a secondary channel using argon plasma coagulation ( Fig. 2 **a** ). Subsequently, contrast imaging was performed using a duodenoscope (TJF-Q290V, Olympus, Japan), confirming complete stent obstruction ( Fig. 2 **b, c** ). Despite multiple attempts, a 0.025-in. angle-type guidewire could not be passed through the obstructed site ( Fig. 2 **d** ).

**Fig. 2:**
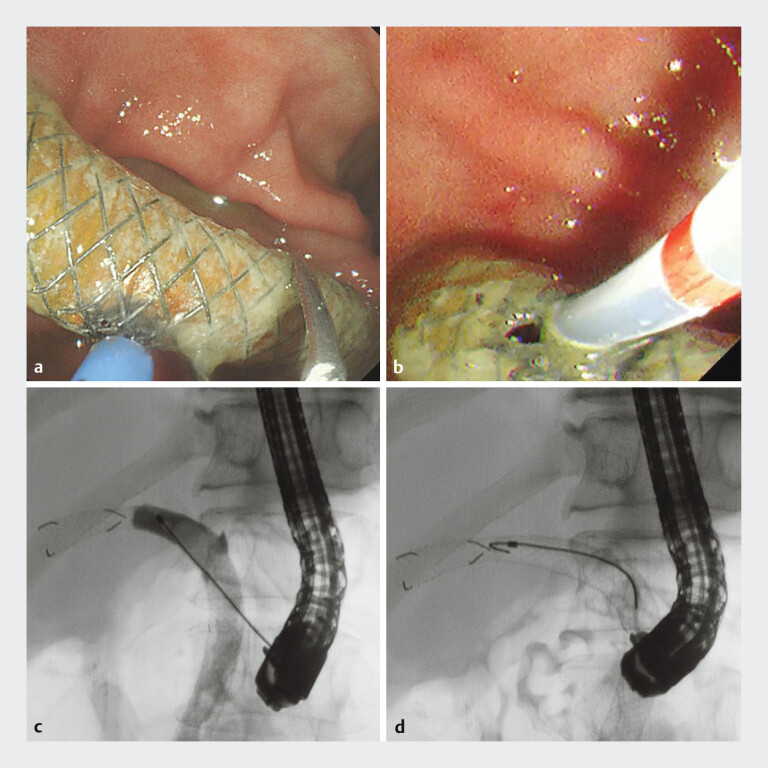
Reintervention using an endoscopic retrograde cholangiopancreatography (ERCP) catheter. **a** The SEMS trimmed using argon plasma coagulation. **b** The ERCP catheter is inserted into the SEMS. **c** Cholangiography confirmed biliary obstruction. **d** Attempts at guidewire passage were unsuccessful.

Peroral cholangioscopy (Spyglass DS, Boston Scientific, Marlborough, USA) was advanced through the SEMS, revealing complete obstruction of the uncovered segment by hyperplastic tissue ( Fig. 3 **a, b** ). A 0.025-in. straight-type guidewire was successfully navigated into the common bile duct under direct cholangioscopic visualization ( Fig. 4 **a, b** ). Balloon dilation of the obstructed site was then performed, followed by the placement of a plastic stent across the obstruction ( Fig. 4 **c, d** ).

**Fig. 3:**
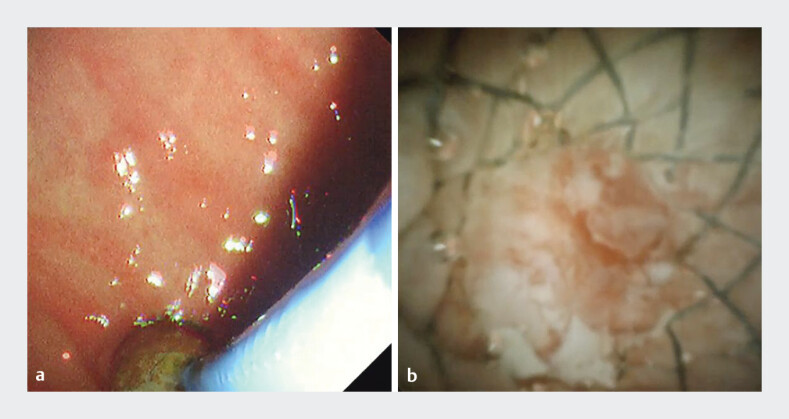
**a** A cholangioscope introduced into the SEMS. **b** Uncovered segment of the SEMS is completely occluded by hyperplastic tissue.

**Fig. 4:**
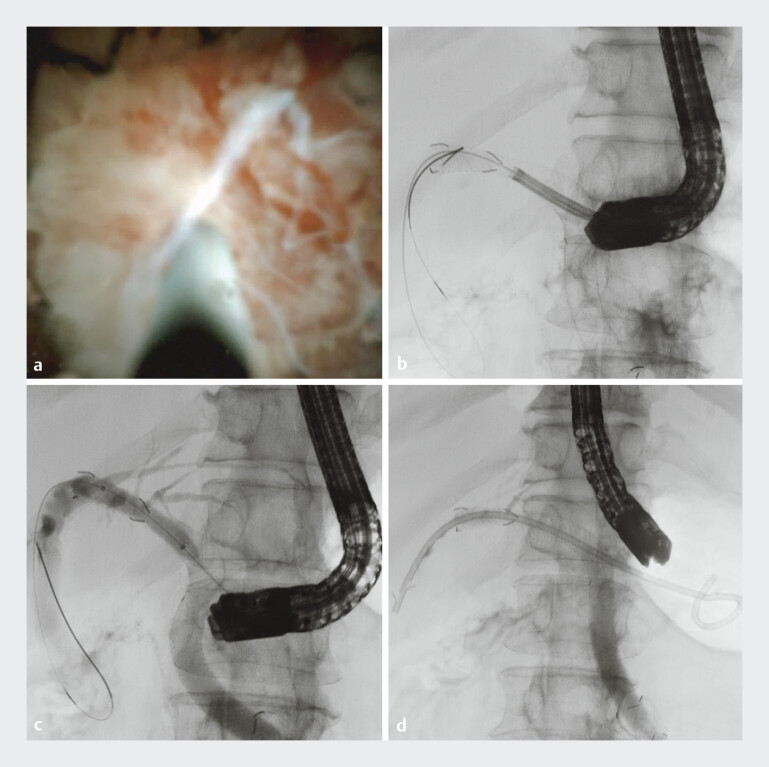
Reintervention using peroral cholangioscopy. **a, b** Guidewire passage was successfully performed under direct cholangioscopic visualization. **c** Balloon dilation at the obstruction site. **d** Plastic stent placed in the bile duct.

Peroral cholangioscopy-guided reintervention for SEMS obstruction after EUS-HGS can be an effective approach, particularly in cases where conventional guidewire passage is challenging.

Endoscopy_UCTN_Code_TTT_1AS_2AH

## Notes

Endoscopy E-Videos https://eref.thieme.de/e-videos
*E-Videos* is an open access online section of the journal *Endoscopy* , reporting on interesting cases and new techniques in gastroenterological endoscopy. All papers include a high-quality video and are published with a Creative Commons CC-BY license. Endoscopy E-Videos qualify for HINARI discounts and waivers and eligibility is automatically checked during the submission process. We grant 100% waivers to articles whose corresponding authors are based in Group A countries and 50% waivers to those who are based in Group B countries as classified by Research4Life (see: https://www.research4life.org/access/eligibility/ ). This section has its own submission website at https://mc.manuscriptcentral.com/e-videos .
